# Longitudinal Diffusion Tensor Imaging-Based Assessment of Tract Alterations: An Application to Amyotrophic Lateral Sclerosis

**DOI:** 10.3389/fnhum.2017.00567

**Published:** 2017-12-05

**Authors:** Dobri Baldaranov, Andrei Khomenko, Ines Kobor, Ulrich Bogdahn, Martin Gorges, Jan Kassubek, Hans-Peter Müller

**Affiliations:** ^1^Department of Neurology, University of Regensburg, Regensburg, Germany; ^2^Department of Neurology, University of Ulm, Ulm, Germany

**Keywords:** magnetic resonance imaging, diffusion tensor imaging, neurodegeneration, neurodegenerative disease, DTI metrics

## Abstract

**Objective**: The potential of magnetic resonance imaging (MRI) as a technical biomarker for cerebral microstructural alterations in neurodegenerative diseases is under investigation. In this study, a framework for the longitudinal analysis of diffusion tensor imaging (DTI)-based mapping was applied to the assessment of predefined white matter tracts in amyotrophic lateral sclerosis (ALS), as an example for a rapid progressive neurodegenerative disease.

**Methods**: DTI was performed every 3 months in six patients with ALS (mean *(M)* = 7.7; range 3 to 15 scans) and in six controls (*M* = 3; range 2–5 scans) with the identical scanning protocol, resulting in a total of 65 longitudinal DTI datasets. Fractional anisotropy (FA), mean diffusivity (MD), axonal diffusivity (AD), radial diffusivity (RD), and the ratio AD/RD were studied to analyze alterations within the corticospinal tract (CST) which is a prominently affected tract structure in ALS and the tract correlating with Braak’s neuropathological stage 1. A correlation analysis was performed between progression rates based on DTI metrics and the revised ALS functional rating scale (ALS-FRS-R).

**Results**: Patients with ALS showed an FA and AD/RD decline along the CST, while DTI metrics of controls did not change in longitudinal DTI scans. The FA and AD/RD decrease progression correlated significantly with ALS-FRS-R decrease progression.

**Conclusion**: On the basis of the longitudinal assessment, DTI-based metrics can be considered as a possible noninvasive follow-up marker for disease progression in neurodegeneration. This finding was demonstrated here for ALS as a fast progressing neurodegenerative disease.

## Introduction

Diffusion tensor imaging (DTI) allows analysis of the structural connectivity in neurodegenerative diseases such as amyotrophic lateral sclerosis (ALS; Bede and Hardiman, 2014; Agosta et al., 2015; Kassubek and Müller, 2016), Alzheimer’s disease (Teipel et al., 2014), and Parkinsonism (Cochrane and Ebmeier, 2013; Meijer et al., 2013). DTI can quantify the integrity of large white matter tracts *in vivo* using metrics such as fractional anisotropy (FA), mean diffusivity (MD), and axial (AD) and radial diffusivity (RD; Pierpaoli and Basser, 1996; Le Bihan et al., 2001). The statistical analysis can be performed by various approaches, e.g., whole brain-based spatial statistics (WBSS; Müller and Kassubek, 2013) or tract-based quantification by analyzing DTI metrics along tract systems (Sarica et al., 2017). Well established techniques in this field are tract based spatial statistics (TBSS—Smith et al., 2006), tracts constrained by underlying anatomy (TRACULA—Sarica et al., 2014), or tractwise fractional anisotropy statistics (TFAS—Müller et al., 2007b). An overview of standardized DTI analysis tools is given in Soares et al. (2013).

Specifically, the use of DTI has substantially improved the understanding of the *in vivo* cerebral and spinal neuropathology of the neurodegenerative disorder ALS (Turner, 2011; Turner et al., 2011, 2012; Filippi et al., 2015) as a rapidly progressive neurodegenerative disease with a well-defined neuroanatomical propagation pattern (Braak et al., 2013; Brettschneider et al., 2013; Jucker and Walker, 2013). The extensive application to the study of ALS has undoubtedly improved the understanding of disease pathophysiology and is likely to have a role in the identification of potential biomarkers of disease progression (Agosta et al., 2010). The recently introduced neuropathological staging system in ALS (Braak et al., 2013; Brettschneider et al., 2013, 2015) has already been transferred to a DTI-based *in vivo* imaging concept, indicating that ALS may disseminate in regional patterns (Kassubek et al., 2014; Müller et al., 2016). The initially affected CNS tract structure is the corticospinal tract (CST), as the correlate of histopathological ALS-stage 1 (Kassubek et al., 2014; Müller et al., 2016).

To this end, longitudinal studies in neurodegeneration are superior to cross-sectional studies in characterizing specific disease phenotypes and genotypes (Schuster et al., 2015). Previous longitudinal magnetic resonance imaging (MRI) studies in ALS have already been applied to subject groups with follow-up visits and reported FA reduction in the CST (Cardenas-Blanco et al., 2016; Kassubek et al., 2017). However, longitudinal MRI studies are rare particularly in ALS, due to the strains that MRI data acquisition puts on severely handicapped patients with this fast progressive disease so that longitudinal studies usually include a baseline and one or two follow-up scans (Zhang et al., 2011; Keil et al., 2012; Kwan et al., 2012; Abhinav et al., 2014; Menke et al., 2014; Steinbach et al., 2015; Cardenas-Blanco et al., 2016). In the current study, an investigation by DTI-based metrics in ALS patients with up to 14 follow-up scans was performed.

The aim of this study was to correlate *in vivo* imaging markers with the clinical performance (ALS-FRS-R score) over time in order to assess the DTI-based correlates of clinical progression and, mechanistically, to analyze the biomarker potential of DTI to assess disease progression. The hypothesis of this study was that DTI metrics alterations in ALS related tract systems correlate to parameters of clinical progression and thus might be used as an additional marker for disease progression.

## Materials and Methods

### Subjects and Scanning Protocol

Sixty-five longitudinal DTI data sets from six ALS patients and six healthy subjects were recorded with the identical scanning protocol after informed consent; the average time interval between the scans was 3 months (interquartile range 3–4 months). Patients were assessed only if they felt capable for MRI examination. The mean follow-up time was 26 months (range 9–48 months). Patients were 5 males and 1 female (mean age 43 years, range 26–60), and controls were 5 males and 1 female (mean 40 years, range 24–54). All ALS patients were diagnosed according to revised El Escorial criteria (Ludolph et al., 2015) and had spinal onset (3 with upper limb). Mean ALS-FRS-R was initially 41 (range 22–48), mean disease duration from diagnosis was 5 months (range 1–14 months), mean time from first symptom was 18 months (range 8–41 months). For the assessment of the clinical condition, the revised ALS functional rating scale (ALS-FRS-R; Cedarbaum et al., 1999) was determined each month. All ALS patients had received longterm G-CSF (Zhang et al., 2009; Pollari et al., 2011) after informed consent on a named patient basis (compassionate use, local ethics committee of the University of Regensburg, project # 15-101-0106). The details about the participants and the participants’ toxicology can be found in Grassinger et al. (2014). All subjects gave written informed consent in accordance with the Declaration of Helsinki. Scan statistics and subjects characterization are summarized in Table 1.

**Table 1:** Baseline characteristics of patients and controls.

Subject	Age/years	Scans	ALS-FRS-R	Site of onset	Time from diagnosis to baseline MRI (months)	Time from first symptom to baseline MRI (months)	ALS-FRS-R rate of decline/(% per year)	Duration of follow-up
ALS 01	40–50	5	48	R LE	2	8	13	15
ALS 02	60–70	3	41	L UE	5	12	25	9
ALS 03	50–60	6	44	L LE	1	14	21	20
ALS 04	20–30	15	26	L UE	14	23	5	48
ALS 05	20–30	9	45	L UE	2	8	13	36
ALS 06	40–50	8	44	R LE	4	41	10	27
control 01	30–40	5	na	na	na	na	na	35
control 02	30–40	4	na	na	na	na	na	27
control 03	20–30	3	na	na	na	na	na	24
control 04	50–60	2	na	na	na	na	na	5
control 05	40–50	2	na	na	na	na	na	4
control 06	50–60	2	na	na	na	na	na	4
*p* (*t*-test)	0.7	na	na	na	na	na	na	0.3

*ALS, amyotrophic lateral sclerosis; m, male; f, female; ALSFRS-R, ALS Functional Rating Scale Revised; R LE, right lower extremity; L LE, left lower extremity; R UE, right upper extremity; L UE, left upper extremity; MRI, magnetic resonance imaging, time from diagnosis was obtained according to the referral letter or own diagnostics, the patient ware asked about the time of first symptoms*.

Scanning was performed on a 1.5 Tesla clinical scanner (Aera, Siemens Medical, Erlangen, Germany). The DTI protocol consisted of 3 × 21 volumes (25 slices, 128 × 128 pixels, slice thickness 5.0 mm, in-plane pixel size 1.8 × 1.8 mm^2^), representing 20 gradient directions (GD) and one scan with gradient 0 (b0); repetition time (TR) was 3500 ms, echo time (TE) was 83 ms, and the b-value was 1000 s/mm^2^, acquisition time was 7 min.

### Data Analysis

**Figure 1:**
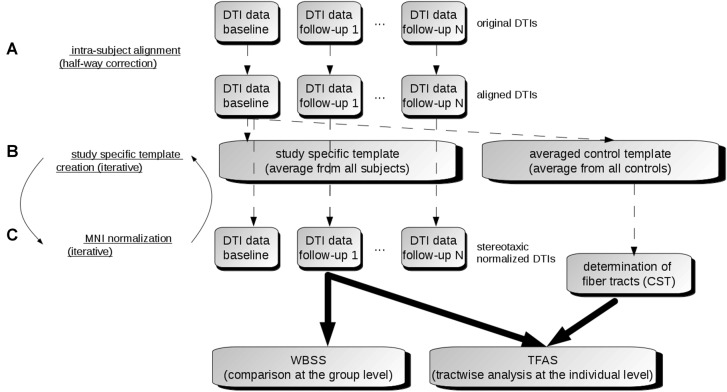
Data analysis scheme. **(A)** In order to obtain a common coordinate frame, all diffusion tensor imaging (DTI) data (b0) were aligned to baseline data. From these b0, a subject-specific template was created for each subject separately. In the next step, b0 of baseline and follow-ups were aligned to the subject-specific template. **(B)** After creation of a study-specific template in the Montreal Neurological Institute (MNI) coordinate frame, DTI data of all visits were stereotaxically normalized **(C)**. DTI metrics maps were calculated from normalized DTI data. Then, the voxelwise statistical comparison between the patients and the control group was performed. After averaging controls’ data sets, fiber tracts were calculated from this averaged data set. Finally, tractwise fractional anisotropy statistics (TFAS) was applied.

The DTI analysis software *Tensor Imaging and Fiber Tracking* (TIFT; Müller et al., 2007a) was used for the data processing. For an overview of the analysis procedure, a schematic description is provided in Figure 1.

### Alignment of Individual Longitudinal Scans

In a first step, b0 maps of longitudinal scans were aligned to the b0 map of the baseline scan for all individuals separately by a conjugate simplex fitting procedure, thus minimizing the intensity differences. In a second step, an average b0 map was calculated for each individual from all aligned b0 maps, and then all b0 maps (including the baseline scan) were aligned to that b0 template. Thus, the bias of the baseline scan (Menke et al., 2014) was minimized (Figure 1A).

### Stereotaxic Normalization and DTI Metrics

Spatial normalization to the Montreal Neurological Institute (MNI) stereotaxic standard space (Brett et al., 2002) was performed by creating a study-specific (b0)-template and FA-template in an iterative manner (Müller and Kassubek, 2013; Figure 1B). DTI-based maps, i.e., FA, AD, MD, RD and the ratio AD/RD, were calculated from these MNI normalized data sets. FA is a summary measure of microstructural integrity. While FA is highly sensitive to microstructural changes, it is less specific to the type of change. MD is an inverse measure of the membrane density, AD tends to be variable in WM changes and axonal injury, and RD increases in WM with dysmyelination. Changes in the axonal diameters or density may also influence RD (Song et al., 2002). The ratio AD/RD correlates with white matter disruption in pathological states whereas these data suggest myelination and/or inflammation in gray matter (Wang et al., 2015). Finally, these DTI metrics maps were smoothed with an 8 mm full width at half-maximum Gaussian filter in order to achieve a good balance between sensitivity and specificity (Unrath et al., 2010; Rosenbohm et al., 2016).

### Whole Brain-Based Spatial Statistics (WBSS)—Comparison at the Group Level

WBSS for all DTI metrics was performed as a voxelwise comparison by Student’s *t*-test. Voxels with FA values below 0.2 were not considered for statistical analysis as cortical gray matter shows FA values up to 0.2 (Kunimatsu et al., 2004). Results were corrected for multiple comparisons at *p* < 0.05 using the false-discovery-rate (FDR) algorithm (Genovese et al., 2002). Clustering procedure for further reduction of type I and type II errors was applied with a threshold cluster size of 512 voxels, corresponding to a sphere with a radius of approximately two acquisition voxels (Figure 1C).

### Fiber Tracking and Tractwise Fractional Anisotropy Statistics (TFAS)

For fiber tracking (FT), an averaged DTI data set was calculated from control data sets by arithmetic averaging of the MNI transformed data while preserving directional information of individual data sets (Müller et al., 2009). This averaged control DTI data set was then used to identify the tract structures with a seed-to-target approach for which seed and target region had a radius of 10 mm each, defining a tract of interest (TOI). For the FT technique, a modified deterministic streamline tracking approach was used (Müller et al., 2007b). Parameters for FT were an FA-threshold of 0.2 and an Eigenvector scalar product threshold of 0.9. In a consecutive step, the technique of TFAS (Müller et al., 2009, 2012) was applied for quantification by use of a TOI-based selection of FA values underlying the FT (Figure 1C). TFAS has originally been developed for FA analysis and has been extended to analyze also further DTI metrics as AD, RD, MD and the ratio AD/RD (Rosenbohm et al., 2016).

TFAS was performed for each scan of each individual, and the changes of averaged DTI metrics’ values were used to calculate the progression rate (in % per year) by

(1) $$P_{\text{DTI}}=-s/0.365*12$$

where 0.365 is the mean FA value of controls in the CST and *s* is the elevation obtained by fitting a regression line. The progression rate (in % per year) for ALS-FRS-R was defined by

(2) $$P_{\text{ALS}–\text{FRS}–\text{R}}=-s/48*12$$

In order to provide an estimation of the reproducibility, the coefficient of variation was determined for the different DTI metrics of controls.

## Results

### Mapping of the Corticospinal Tract by Fiber Tracking

**Figure 2:**
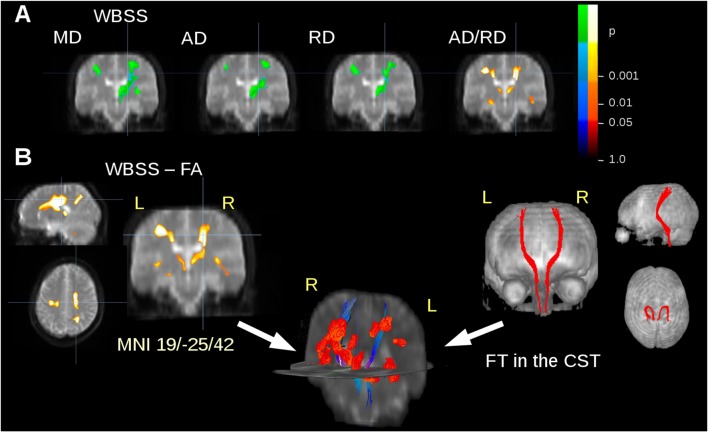
DTI metrics alterations at the group level. **(A)** Whole brain-based spatial statistics (WBSS) of the comparison amyotrophic lateral sclerosis (ALS) patients vs. controls: results clusters of mean diffusivity (MD), axial diffusivity (AD), radial diffusivity (RD), and the ratio AD/RD for coronal slice at *y* = −25. **(B)** Results clusters for Fractional anisotropy (FA) maps (as an example representative for all calculated DTI metrics) and projectional views of fiber tracking (FT) of the corticospinal tract (CST). Overlap of fiber tracts along the CST and FA differences at the group level in 3-D view. Increase is displayed in cold colors, decrease is displayed in hot colors.

The ALS patients showed ALS-associated white matter alterations in general agreement with previous studies (e.g., Salat et al., 2005; Müller et al., 2016), i.e., an FA decrease along the CST. In order to avoid an unequal weighting in the statistical analysis for the subjects with five or more scans compared to subjects with lower scan numbers, WBSS results were limited to a maximum of two scans from each subject. MD, AD and RD showed an increase in regions along the CST, while FA and the ratio AD/RD showed a decrease in these areas (Figure 2A). For FT along the CST, seed and target MNI coordinates were ±22/−20/14 and ±24/−30/50, respectively. Figure 2B shows an overlay of cross-sectional FA results clusters and fiber tracts.

### Longitudinal Screening of FA-Values in the CST

**Figure 3:**
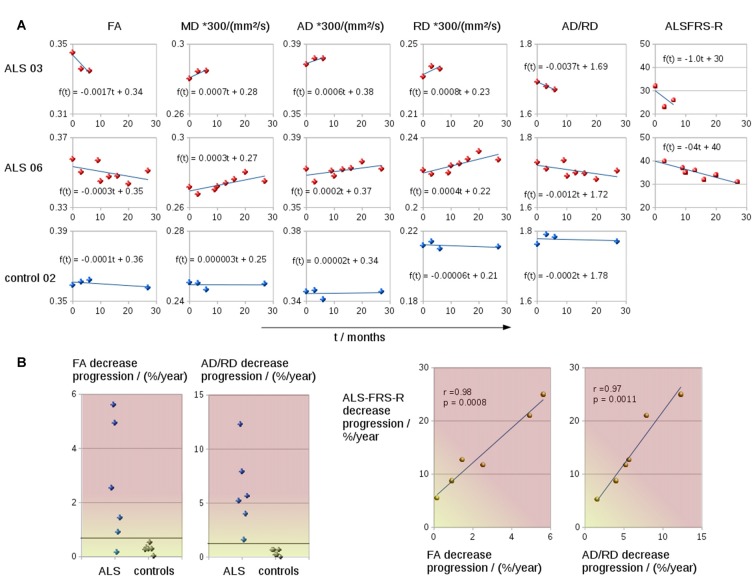
**(A)** Charts of DTI metrics (FA, MD, AD, RD, the ratio AD/RD) and ALS-FRS-R alterations during the course of disease of two representative ALS patients (one fast progressor, one slow progressor) and one representative control. **(B)** FA and AD/RD decrease progression in the CST for six ALS patients and six controls. FA and AD/RD decrease progression correlated significantly with ALSFRS-R decrease.

The coefficient of variation demonstrated a high reproducibility in DTI metrics (Table 2). Figure 3A shows exemplary charts of DTI metrics alterations (AD, FA, MD, RD, AD/RD) and ALS-FRS-R decrease progression over time from two ALS patients and one control. FA and AD/RD decrease progression were less than 0.4% per year for controls (compare Salat et al., 2005), whereas in ALS patients, FA decrease progression ranged between 0.2% to 5.6% per year and AD/RD decrease progression ranged between 1.6% to 12.3% per year. The remaining DTI metrics (AD, MD, RD) partially overlapped between patients and controls (Table 3). Figure 3B shows the different patterns of the FA and AD/RD decrease progression rates in ALS patients and controls.

**Table 2:** Arithmetically averaged standard deviation and coefficient of variation (COV) as a reproducibility measure of controls’ DTI metrics axial diffusivity (AD), fractional anisotropy (FA), mean diffusivity (MD), radial diffusivity (RD), and AD/RD.

DTI-metric	AD/(mm^2^/s)	FA	MD/(mm^2^/s)	RD/(mm^2^/s)	AD/RD
Averaged mean	102.8	0.359	74.1	61.3	1.77
Averaged standard deviation	0.49	0.00095	0.21	0.34	0.036
Averaged COV	0.0048	0.0026	0.0028	0.0055	0.0020

**Table 3:** Arithmetically averaged progression rates (in %/year) for ALS patients and controls (mean ± standard deviation) for DTI metrics—axial diffusivity (AD), fractional anisotropy (FA), mean diffusivity (MD), radial diffusivity (RD) and AD/RD.

Progression rates (in %/year)	ALS patients	Range (ALS patients)	Controls	Range (controls)	Correlation to ALS-FRS-R progression
FA (decrease)	2.6 ± 2.2	0.2–5.6	0.3 ± 0.2	0.0–0.5	0.98 (*p* = 0.0008*)
MD (increase)	−1.9 ± 1.5	−4.6 to −0.6	−0.08 ± 0.05	−0.2 to 0.0	−0.65 (*p* = 0.2)
AD (increase)	−2.0 ± 2.2	−6.2 to −0.2	−0.1 ± 0.6	−0.8 to 0.6	−0.47 (*p* = 0.3)
RD (increase)	−2.2 ± 1.2	−4.3 to −1.2	−0.1 ± 0.8	−1.0 to 1.0	−0.66 (*p* = 0.2)
AD/RD (decrease)	6.1 ± 3.7	1.6–12.3	0.4 ± 0.3	0.0–0.7	0.97 (*p* = 0.0011*)
ALS-FRS-R (decrease)	14 ± 7	6–25	n.a.	n.a.	n.a.

*Significant correlation.

Both FA and AD/RD decrease progression correlated significantly with ALS-FRS-R decrease progression (*r* = 0.98, *p* = 0.0008 and *r* = 0.97, *p* = 0.0011, respectively; Table 3). In this sample of ALS patients, four subjects showed a slower progression rate of less than 2% per year, whereas two ALS patients showed higher progression rate between 3% and 6% per year (Figure 3B). These latter two patients also showed a rapid worsening in ALS-FRS-R between 10 and 15 points per year.

## Discussion

In this methodological study, a longitudinal DTI analysis concept was applied to assess the disease progression in ALS patients with a high number of follow-up scans. Patients with ALS showed alterations along the CST correlating with the clinical progression (ALS-FRS-R decline). Therefore, this study provides a framework for the analysis of longitudinal DTI data in order to map progressive neurodegeneration. ALS was chosen as an example for a neurodegenerative disease with a generally rapid progression in which a prominent tract structure, i.e., the CST, is affected in the early course of the disease, corresponding to neuropathological stage 1 (Braak et al., 2013; Kassubek et al., 2014).

In the small sample of subjects, different progression trends could be identified, i.e., controls showed DTI metrics alterations that were in line with standard ageing-related alterations (Salat et al., 2005; Lim et al., 2015), while ALS patients showed different progression types in terms of alterations along the CST. From the clinical viewpoint, the patients (who all had spinal ALS onset) were also heterogeneous in their clinical presentations. In patients with faster progression, both clinical disease severity according to ALS-FRS-R and DTI metrics alterations showed a marked decline during the observation period. The effect of G-CSF on ALS-progression rate was not the topic of this imaging study and will be the subject of a separate data analysis. In the same line, we did not perform a genetic analysis in this imaging oriented context, all the more since there was no obvious evidence of family history in all individuals. In future studies, additional analyses like the vascular density index might be used.

The present study is limited by the number of subjects but can be considered as an indicator for the potential of the methodology. As an advantage, the identification of tract structures by DTI techniques can be considered rather operator independent in contrast to a region-of-interest analysis. Further, the methodological approach is independent of age so that age-related changes of the DTI metrics (Salat et al., 2005; Lim et al., 2015) could be neglected for the follow-up interval of about 2 years. A bias of the baseline scan in terms of alignment (Menke et al., 2014) could be assumed, but was minimized by the creation of subject-specific templates. Final progression results in DTI metrics (Figure 3) showed no systematic bias for the baseline scans (see Menke et al., 2014), i.e., the values of the baseline scan fitted the regression line of all DTI metrics values of all scans. The highly significant correlation between DTI metrics alterations (both FA and AD/RD) with ALS-FRS-R decrease might offer an opportunity to assess disease progression with a lower number of patient visits: an accuracy assessment of the disease progression might either be obtained by a higher number of patient visits (with ALS-FRS-R scores at each visit) or by a less frequent number of visits and a determination of disease progression by ALS-FRS-R in combination with DTI metrics.

There are some limitations in the acquisition protocol. The examination was done at 1.5T with 20 directions, 1 b-value, and 5 mm slice thickness. The slice thickness was relatively large for FT. However, we have identified the CST with straightforward superior-inferior directionality with sufficient accuracy. If more subtle fronto-dorsal or lateral tract structures or a mixture of the three directions are to be analyzed, a slice thickness of 5 mm might be too large. However, it could be demonstrated that DTI metrics could be assessed as a technical marker even with such a restricted DTI protocol, which should be available at each clinical scanner and could be run in any routine data acquisition. Moreover, the protocol with a short acquisition time of 7 min was well tolerated by ALS patients also in an advanced clinical condition so that it was possible to acquire this higher number of follow-up scans.

This longitudinal DTI-based analysis of structural tract changes addressed the identification of a straightforward, non-invasive, quantitative *in vivo* biomarker of neurodegeneration which correlates with clinical progression. Potentially, these results will encourage future longitudinal studies in neurodegenerative diseases. Here, the applied methodology might be used to study other tract structures that are specific for the given neurodegenerative disease, e.g., Braak stages in Alzheimer’s Disease (Braak and Braak, 1991) or Braak stages in Parkinson’s Disease (Braak and Del Tredici, 2009).

## Author Contributions

DB and AK: substantial contribution to the conception of the study, data acquisition, data analysis, critical revision of the manuscript, final approval of the version to be published. IK: substantial contribution to the data acquisition, critical revision of the manuscript, final approval of the version to be published. UB: substantial contribution to the conception and design of the study and the interpretation of the data, critical revision of the manuscript, final approval of the version to be published. MG: substantial contribution to the data analysis, critical revision of the manuscript, final approval of the version to be published. JK: substantial contribution to the design of the study and the interpretation of the data, critical revision of the manuscript, final approval of the version to be published. H-PM: substantial contribution to the conception of the study and the data analysis, critical revision of the manuscript, final approval of the version to be published.

## Conflict of Interest Statement

The authors declare that the research was conducted in the absence of any commercial or financial relationships that could be construed as a potential conflict of interest.
